# Arctic browning: Impacts of extreme climatic events on heathland ecosystem CO_2_ fluxes

**DOI:** 10.1111/gcb.14500

**Published:** 2018-11-25

**Authors:** Rachael Treharne, Jarle W. Bjerke, Hans Tømmervik, Laura Stendardi, Gareth K. Phoenix

**Affiliations:** ^1^ Department of Animal and Plant Sciences The University of Sheffield Sheffield UK; ^2^ Norwegian Institute for Nature Research High North Research Centre for Climate and the Environment Tromsø Norway; ^3^ Free University of Bozen Bolzano Bolzano Italy

**Keywords:** arctic, browning, *Calluna vulgaris*, climate change, dwarf shrub, extreme events, snow cover, stress, winter

## Abstract

Extreme climatic events are among the drivers of recent declines in plant biomass and productivity observed across Arctic ecosystems, known as “Arctic browning.” These events can cause landscape‐scale vegetation damage and so are likely to have major impacts on ecosystem CO_2_ balance. However, there is little understanding of the impacts on CO_2_ fluxes, especially across the growing season. Furthermore, while widespread shoot mortality is commonly observed with browning events, recent observations show that shoot stress responses are also common, and manifest as high levels of persistent anthocyanin pigmentation. Whether or how this response impacts ecosystem CO_2_ fluxes is not known. To address these research needs, a growing season assessment of browning impacts following frost drought and extreme winter warming (both extreme climatic events) on the key ecosystem CO_2_ fluxes Net Ecosystem Exchange (NEE), Gross Primary Productivity (GPP), ecosystem respiration (*R*_eco_) and soil respiration (*R*_soil_) was carried out in widespread sub‐Arctic dwarf shrub heathland, incorporating both mortality and stress responses. Browning (mortality and stress responses combined) caused considerable site‐level reductions in GPP and NEE (of up to 44%), with greatest impacts occurring at early and late season. Furthermore, impacts on CO_2_ fluxes associated with stress often equalled or exceeded those resulting from vegetation mortality. This demonstrates that extreme events can have major impacts on ecosystem CO_2_ balance, considerably reducing the carbon sink capacity of the ecosystem, even where vegetation is not killed. Structural Equation Modelling and additional measurements, including decomposition rates and leaf respiration, provided further insight into mechanisms underlying impacts of mortality and stress on CO_2_ fluxes. The scale of reductions in ecosystem CO_2_ uptake highlights the need for a process‐based understanding of Arctic browning in order to predict how vegetation and CO_2_ balance will respond to continuing climate change.

## Graphical abstract

Extreme climatic events are among the drivers of vegetation damage and decline observed across Arctic ecosystems in recent years; termed “Arctic browning.” Although these events can cause landscape‐scale vegetation damage, their impacts on ecosystem CO_2_ balance are little understood. Here, it is demonstrated that these events can have major impacts on CO_2_ balance, considerably reducing the carbon sink capacity of the ecosystem across the growing season. These impacts can be similar when associated with shoot mortality, or with shoot stress responses.

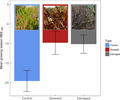

## 1 INTRODUCTION

The Arctic is warming twice as fast as the global average, with the most rapid temperature increases occurring during the winter months (AMAP, 2017; Richter‐Menge, Overland, Mathis, & Osborne, 2017). Warmer winters, in combination with greater temperature variability, are associated with increasingly frequent winter extreme climatic events that cause major disturbance in Arctic ecosystems (Beniston et al., 2007; Graham et al., 2017; Johansson, Pohjola, Jonasson, & Callaghan, 2011; Vikhamar‐Schuler et al., 2016).This disturbance includes severe vegetation damage and mortality, often at landscape or greater scales (Bjerke et al., 2014; Bokhorst, Bjerke, Tømmervik, Callaghan, & Phoenix, 2009; Phoenix & Bjerke, 2016).

Such extreme events are therefore among the key drivers of “Arctic browning,” the term used to describe the declining biomass and productivity observed at regional to pan‐Arctic scales in recent years (Epstein, Bhatt, & Raynolds, 2015, 2016; Miles & Esau, 2016; Phoenix & Bjerke, 2016). Only one previous study has made a full assessment of CO_2_ fluxes following browning driven by an extreme climatic event (Parmentier et al., 2018). While this work in a northern peatland indicated a 12% reduction in GPP, full assessment of this event's effect on eddy covariance CO_2_ fluxes was challenging due to large inter‐annual variability in summer climate. Similarly, a single time‐point measurement of GPP following browning driven by simulated extreme winter warming found a reduction of more than 50% (Bokhorst, Bjerke, Street, Callaghan, & Phoenix, 2011). However, despite the scale of the impacts suggested by this snapshot of peak season CO_2_ fluxes, measurements taken across the growing season are crucial to our understanding of how extreme events are impacting CO_2_ balance. This is of particular importance in Arctic ecosystems where the growing season, and therefore the window of opportunity for substantial CO_2_ fixation, is short and dynamic (Callaghan et al., 2011; Larsen, Ibrom, Jonasson, Michelsen, & Beier, 2007). In addition, while previous work has focussed on vegetation mortality driven by extreme events, sub‐lethal stress (indicated by high, persistent anthocyanin pigmentation, and an associated deep red‐burgundy coloration) is also often observed following extreme events. Although this stress response has been observed in dwarf shrub ecosystems from boreal to High Arctic latitudes (Bjerke et al., 2017), it is not known whether it impacts shoot‐level and ecosystem CO_2_ fluxes.

Extreme event drivers of Arctic browning may be climatic, biological (e.g., defoliating insect outbreak) or physical (e.g., fire) (Bjerke et al., 2017; Bokhorst et al., 2008; Jepsen et al., 2013; Mack et al., 2011; Phoenix & Bjerke, 2016). Among the most damaging and frequently observed climatic extreme events is frost drought (Bjerke et al., 2014). Frost drought events arise from loss or absence of a protective snow layer in winter, either through low snow fall, anomalous winter warmth causing substantial snow melt or wind displacement. In these conditions, since soils remain frozen or near‐frozen, plant water uptake is limited so that transpiration of exposed vegetation can result in desiccation injury (Sakai & Larcher, 1987; Tranquillini, 1982). This process is accelerated where exposure occurs in combination with high winds or irradiance (Bjerke et al., 2014, 2017; Hadley & Smith, 1989, 1986). Damage following frost drought is readily visible as partial to complete mortality of above ground shoots (i.e., “browning”), and hence, this damage is similar to that caused by extreme winter warming events (another extreme event driver of browning), when winter snow melt exposes vegetation to unseasonably warm temperatures sufficient to initiate premature dehardening, subsequently resulting in freeze damage on return of sub‐zero winter temperatures (Bokhorst et al., 2010; Phoenix & Lee, 2004). It is of concern, therefore, that the frequency of mid‐winter thaw episodes, the central characteristic of both extreme winter warming and frost drought events, may as much as double during this century in some Arctic regions (Johansson et al., 2011; Vikhamar‐Schuler et al., 2016). These extreme events may therefore have an increasing influence over high‐latitude vegetation.

Already, individual events such as these are causing landscape‐level (>1,000 km^2^) reductions in vegetation greenness (Bokhorst et al., 2009; Hansen et al., 2014; Phoenix & Bjerke, 2016). At the same time, increased browning trends (to which trend climate change, such as reduced summer warmth and delayed snow melt, also contribute) have been observed across the Arctic (Bhatt et al., 2013; Epstein et al., 2015; Epstein et al., 2016; Bieniek et al., 2015). The severity of browning from extreme events can be considerable, as demonstrated when multiple extreme events in 2012, including frost drought and extreme winter warming, reduced Normalised Difference Vegetation Index (NDVI, a measure of greenness) to the lowest levels ever recorded across the Nordic Arctic Region (Bjerke et al., 2014).

These large‐scale impacts reflect shoot mortality (Bokhorst et al., 2011, 2009), but likely also the sub‐lethal stress response indicated by high, persistent anthocyanin pigmentation, that has nonetheless received limited attention to date (Bjerke et al., 2017). A plot‐level assessment of areas affected by extreme climatic events from boreal to High Arctic latitudes found this visible stress response affecting up to 75% of *Calluna vulgaris *shoots, in addition to high levels of mortality observed in shoots of the evergreen shrubs *Calluna vulgaris* and *Cassiope tetragona* (up to 60% and 50%, respectively) (Bjerke et al., 2017). Anthocyanin pigments fulfil a huge diversity of functions in plants; in addition to being associated with discrete developmental stages (such as autumn senescence in deciduous trees), they are involved in responses to a range of stressors including high light levels, cold temperatures and herbivory (Gould, 2004; Landi, Tattini, & Gould, 2015). Accumulation of anthocyanin pigments in leaves during and shortly after snow melt is therefore common in many upland or high‐latitude species (Oberbauer & Starr, 2002). However, since synthesis of anthocyanins incurs a metabolic cost, and their presence reduces light capture by chlorophyll (Burger & Edwards, 1996; Steyn, Wand, Holcroft, & Jacobs, 2002), their accumulation is typically transient (Chalker‐Scott, 1999; Mac Arthur & Malthus, 2012). In contrast, the unusually strong anthocyanin pigmentation observed after frost drought and extreme winter warming persists through much, or even all, of the growing season (personal observation by R. Treharne).

Predicting the future impact of climate change in Arctic ecosystems will require not only quantification of the impacts of browning on ecosystem CO_2_ fluxes, but also mechanistic insights into the processes driving these. For example, changes in ecosystem respiration may be due to altered shoot‐level respiration, or to changes in below‐ground processes such as decomposition, which might be expected to accelerate following higher litter inputs or altered resource allocation following browning (Dahl et al., 2017; Parmentier et al., 2018), or decelerate due to reduced below‐ground carbon allocation (Parker et al., 2017). This process‐based knowledge is critical to building Arctic browning into climate and vegetation models, many of which currently assume an arbitrary level of greening across the Arctic (Pearson et al., 2013).

To address these needs, we report the first detailed assessment of key ecosystem CO_2_ fluxes across the growing season in a sub‐Arctic heathland dominated by *Calluna vulgaris* in northern Norway, quantifying the impacts of both shoot mortality and visible stress following exposure to extreme winter conditions through direct comparison of affected and unaffected vegetation. Plot‐level ecosystem CO_2_ and soil fluxes were measured at plots dominated by either shoot mortality (“damage”), dark red anthocyanin pigmentation (“stress”) or green, healthy vegetation. Structural Equation Modelling (SEM) of these fluxes and plot‐level measures of vegetation greenness, supported by additional measurements of shoot‐level responses (growth, photosynthesis and respiration) and decomposition rates, facilitated more detailed understanding of CO_2 _flux changes associated with damage and stress, and the mechanisms underlying these. Finally, emergent relationships between CO_2_ fluxes and overall browning were used to estimate the upscaled impact on CO_2_ fluxes across the site.

It was hypothesized that (a) NEE, GPP and *R*_eco_ would be reduced both in plots dominated by a visible stress response and, to a greater degree, in those dominated by shoot mortality, compared to green control plots; (b) reductions in these fluxes would diminish throughout the growing season (due to recovery and resprouting of evergreens and leaf‐out of herbaceous species), most rapidly between early and peak season, and more rapidly in plots primarily exhibiting stress compared to those exhibiting shoot mortality; (c) reductions in plot‐level CO_2_ fluxes would be associated with reduced shoot growth and slower decomposition rates; (d) ecosystem CO_2_ fluxes would correlate negatively with overall browning throughout the growing season.

## 2 MATERIALS AND METHODS

### 2.1 Study area

Field work was undertaken in 2016 in sub‐Arctic heathland on the archipelago of Lofoten in northern Norway, at Storfjord (N 68˚ 9' 26.5"E 13˚ 45' 13.5"). This maritime, comparatively southern region, typically experiences a mild winter climate with high levels of snowfall compared to other sub‐Arctic regions (Førland, Benestad, & Flatøy, 2009). Vegetation at the site was dominated by the evergreen shrubs *Calluna vulgaris *(L.) Hull (54% ± 21%) and *Empetrum nigrum *(L.) (37% ± 22%). *Vaccinium *species were also common, and near‐continuous ground cover was provided by feather moss species *Hylocomium splendens* (Hedw.) Schimp and *Pleurozium schreberi *(Brid.) Mitt. The heathland in this ecologically sub‐Arctic (albeit well within the climatological/geographic Arctic, typically defined as the region north of 66˚) is best described as “Empetrum‐Calluna with Vaccinium” according to the definitions laid down by Tveraabak (2004), but according to the Circumpolar Arctic Vegetation Map classification scheme is closest to habitat type code 1.03.3 (Walker et al., 2018). The site was gently undulating with no tree canopy.

A large region of extensive shoot mortality in heathland throughout central and northern Norway, including in the Lofoten Archipelago, was observed following the 2013/2014 winter (Bjerke et al., 2017). This was attributed to frost drought: following an unusually mild December, extremely dry conditions exposed vegetation to cold temperatures and desiccation during January, February and early March 2014. Although some recovery occurred after 2014, significant shoot mortality was still present at smaller scales in many regions following this frost drought event, hence allowing the selection of our study site within this region.

In addition, during the 2015/2016 winter, snow cover and temperature data (accessed from publicly available databases senorge.no and eklima.no) indicate the region experienced a winter warming event, with unusual warmth throughout much of December 2015 (weekly mean temperature elevated by up to 4.2°C compared to normal December mean of −1.2°C), leading to total loss of snow cover. This thaw event was followed by a rapid temperature drop of more than 7°C from 1 to 3 January 2016 (Supporting Information Figure S1). Snow cover remained absent or shallow throughout January. This combination of climatic variables (prolonged warmth and loss of snow cover followed by a marked temperature drop) is unusual, particularly at sufficient severity to trigger an ecological response. However, such events are not unique and have become more common in recent years (Bjerke et al., 2017). In May 2016, around 1 month after spring snow melt, heavy anthocyanin pigmentation (dark red coloration) was observed in widespread *Calluna vulgaris* heathland in multiple locations across the Lofoten region (personal observation by R. Treharne and L. Stendardi).

We selected the Storfjord site based on the presence of both shoot mortality following frost drought in winter 2013/14 and of heavy anthocyanin pigmentation following winter 2015/16, hence allowing us to study and compare both (Supporting Information Figures S2 and S3).

Data were collected in the growing season of 2016, during three measurement periods. Early season measurements were taken between 25 May and 10 June, peak season measurements between 12 and 23 July and late season measurements between 17 and 29 August.

### 2.2 Plot selection

Nineteen 50 × 50 cm plots were selected within a 25 × 60 m study area. We were careful to avoid plots which were in clearly defined hollows, or on hummocks. Cover of dominant ericaceous dwarf shrubs was 75% or more at all plots. Of these plots, six were control plots of healthy, green vegetation, a further six were “damaged plots” with vegetation dominated by past browning (identifiable as dead grey leaves and shoots), and seven were “stressed plots” of vegetation showing high anthocyanin accumulation (identifiable as a dark red pigmentation in leaves and shoots). Of the total 19 plots, 16 were selected for flux measurements (six of damaged, five of stressed and five healthy, green plots). We used 16 rather than all 19 plots for flux measurements simply because of time constraints on gathering flux measurements (see “*Ecosystem CO_2_ flux measurements*”). Plots were marked to enable measurements to be repeated throughout the growing season.

Within each flux plot, percentage cover of browning and dominant species was recorded using a 50 × 50 cm quadrat. Browning was assessed visually by recording percentage cover of green (healthy), grey (shoot mortality from which all photosynthetic pigments have been eliminated, indicating damage is from a previous year), brown (fresh shoot mortality from the winter preceding the measurement season) and dark red vegetation (stress, indicated by high anthocyanin pigmentation).

### 2.3 *Ecosystem CO_2_ flux measurements (NEE, GPP and R*_eco_ *)*

At each flux plot (i.e., 16 of the 19 plots), CO_2_ fluxes were measured using a LiCor LI‐6400 system (LiCor, Bad Homburg, Germany) and a custom, transparent acrylic vegetation chamber, using a similar approach to that of Williams, Street, Wijk, and Shaver (2006) and Street, Shaver, Williams, and Wijk (2007). The chamber was placed on a rigid frame supported by aluminium poles driven into the ground. A seal was provided between the chamber and frame by a rubber gasket, and between the frame and ground surface by a flexible, transparent plastic skirt weighted down with steel chains. PAR was recorded using a LiCor Quantum sensor mounted inside the vegetation chamber. Enclosed volume was determined by using measurements of the height of the frame from the ground across a grid of nine points to calculate volume as four hexahedrons in addition to the chamber volume. Within the chamber, CO_2_ concentration was measured every 2 s for 50 s. Where PAR varied by >15%, measurements were discarded at the analysis stage. Measurements were carried out at 5 light levels (full light, 3 successive levels of shading and dark) in a randomized order using optically neutral shade cloths and tarpaulin.

Light response curves of NEE and GPP were calculated following Street et al. (2007). In brief, CO_2 _concentration over time was converted to CO_2_ flux, allowing the light response of net ecosystem exchange to be modelled as a smooth curve with parallel asymptotes and with a term for ecosystem respiration (*R*_eco_). The *R*_eco_ term was calculated as follows:

$$\text{Net}\;\text{Ecosystem}\;\text{Exchange}=R_{\text{eco}}-\frac{P_{\max}\cdot I}{k+I}$$

where *P*_max_ is the rate of photosynthesis (µmol CO2 m^−2^ s^−1^) under full light, *k* is the half saturation constant of photosynthesis (µmol PAR m^−2^ s^−1^), and *I* is incident PAR. This modelled term was used for *R*_eco_. Subtraction of *R*_eco_ from CO_2_ flux measurements enabled a light response curve of GPP to be fitted, and thus, GPP to be standardized at a PAR of 600 µmol PPFD m^−2^ s^−1^ (GPP_600_). GPP_600_ represents flux at a medium light level and has been used previously to compare GPP between vegetation plots (Shaver et al., 2007). NEE, the net product of *R*_eco_ and GPP, was also standardized at this light level (NEE_600_).

Ground surface temperature was recorded concurrently with CO_2_ concentration data using a temperature probe inserted into the enclosed ground layer vegetation (bryophyte/lichen) or leaf litter. Air temperature was recorded by the LI‐6400. Moss layer and soil surface moisture were recorded after each measurement set using a Delta‐T HH2 moisture meter with a Theta probe (Delta‐T, Cambridge, UK) inserted first at the surface of the moss layer and secondly at the soil surface, following removal of the moss layer. Height of the understory canopy within the plot was measured from the ground at three randomly chosen points following each measurement set.

Plot‐scale measurements of ecosystem CO_2_ fluxes were supported by a smaller set of shoot‐level measurements of photosynthesis and respiration taken at peak season in stressed and controls plots (Supporting Information Section S2).

### 2.4 *Soil respiration (R*_soil_)

An 11‐cm‐diameter soil collar was inserted to a depth of 8 cm adjacent to each 50 × 50 cm flux plot. Vascular vegetation and the green bryophyte layer were clipped to the soil surface before each soil collar was inserted using a serrated cutting blade and mallet. Collars were inserted at the beginning of the early season measurement period and left to stabilize for a minimum of 3 days before measurements were taken (LiCor, 1997). When measurements were not being taken, moss was replaced on top of the collar to minimize soil surface heating and drying due to exposure.

*R*_soil_ measurements were taken using a LiCor LI‐6400 system connected to a LI‐6400–09 soil respiration chamber (LiCor). The 991‐cm^3^ chamber was placed on the soil collar with a foam gasket ensuring a good seal. Before each flux measurement, CO_2_ was scrubbed to 15–20 ppm (dependent on flux) below ambient. Flux was then calculated and recorded as CO_2_ concentration increased from 15 to 20 ppm below to 15–20 ppm above ambient. This cycle was repeated three times for each measurement and an average value calculated.

### 2.5 Shoot growth

Apical shoot growth was also assessed in each plot using retrospective growth analysis. Three live shoots of *Calluna vulgaris* were chosen at random within each plot, and new growth was measured from the shoot tip to that year's bud scar back along the shoot. Growth was measured in this way at peak and late season only, as little shoot growth had taken place at the start of the early season measurement period.

### 2.6 Decomposition

Decomposition and litter stabilization (the proportion of material that becomes recalcitrant during initial decomposition, calculated as the decomposed fraction as a proportion of the chemically labile fraction of material) were assessed using commercially available green and rooibos tea bags, following Keuskamp, Dingemans, Lehtinen, Sarneel, and Hefting (2013) and the Tundra Tea project (2018). In brief, two tea bags of each type were weighed before being buried in separate holes at a depth of 8 cm in an area of vegetation adjacent to and comparable with each plot at the beginning of the early season measurement period. They were then retrieved 3 months later, dried at 70°C for 48 hr and reweighed. Fraction decomposed was calculated incorporating a correction for ambient moisture prior to burial.

### 2.7 Assessment of change in anthocyanin pigmentation

Change in anthocyanin pigmentation was assessed in two apical shoots within each stressed plot and one apical shoot within each control plot; these were tagged at early season and measurements were repeated throughout the growing season. On each shoot, the percentage of total leaf area showing green (healthy), grey (mortality from a previous year), brown (fresh mortality) and dark red (anthocyanin pigmentation) coloration was estimated by eye twice during early season and once during peak and late season measurement periods. The height of these shoots from the ground to the previous year's bud scar was also recorded. Shoots were randomly selected, but only from those shoots within each plot which had not experienced total shoot mortality (i.e., live shoots). This additional shoot‐level damage assessment was used to assess the difference in anthocyanin pigmentation of live vegetation between stressed and damaged plots, and the extent and speed with which pigmented leaf area recovered greenness, or alternatively experienced mortality (i.e., becoming brown), over the course of the growing season in each plot type.

### 2.8 Assessment of site‐level browning

To estimate site‐level browning, two 15‐ to 18‐m transects were completed during each measurement period with percentage cover recorded in a 1 × 1 m area at 3‐m intervals. Percentage cover of stress and damage, as well as cover of dominant species (Supporting Information Table S1), was assessed visually (Supporting Information Section S1).

### 2.9 Statistical analyses and structured equation modelling

Changes in CO_2_ fluxes across the growing season as a whole were assessed using repeated measures ANOVA with linear mixed effects models, incorporating plot number as a random effect. Differences between plot types (damaged, stressed and healthy controls) at each point during the growing season were assessed in more detail using one way ANOVA and Tukey multiple comparison tests.

One way ANOVA and Tukey multiple comparison tests were used to assess differences in shoot growth and litter decomposition. *T* tests were used to assess differences in shoot‐level CO_2_ fluxes between stressed and healthy green shoots. Linear regression was used to assess changes in site‐level (transect) browning and shoot‐level anthocyanin pigmentation and other browning throughout the growing season.

Within each measurement period, linear regression was also used to assess site‐level correlations between percentage cover of total browned vegetation (including stress and damage) and GPP_600_, *R*_eco_ and NEE_600_. This approach was used to look for emergent relationships between total browned vegetation and CO_2_ fluxes and to investigate how differences in the CO_2_ flux impacts of stress and damage might affect these overall relationships. Linear regression of total browning and each CO_2_ flux was compared with multiple regression using separate terms for previous damage (grey) and for fresh damage (brown) combined with stress (dark red). All statistical analyses were carried out in RStudio (R Core Team, 2017).

These analyses were supported by Structural Equation Modelling (SEM) using the lavaan package in RStudio (R Core Team, 2017; Rosseel, 2012). SEM provides a framework for multivariate analysis of networks and paths (Grace et al., 2012; Grace, Anderson, Olff, & Scheiner, 2010). SEM is also a robust approach to provide meaningful analysis of data collected using a survey‐like approach, such as that used here, where variables are likely to be correlated and both direct and indirect effects may be present (Grace, 2006; Graham, 2003). SEM was used here to add weight to conventional statistical tests and to guide inference as to the ecological processes underlying correlations identified through these tests. SEM was initiated by defining an a priori set of theoretically justified paths between variables, guided by the above conventional analyses. This core model was then used to address secondary hypotheses by incorporating additional variables (cover of herbaceous species and canopy height) into the model. SEM included one latent variable. Latent variables are unmeasured constructs, described by correlated indicator variables with an underlying common causal influence. Here, the latent variable “Browning Severity” was used which combined percentage cover of browned vegetation (both mortality and stress) and cover of remaining green ericaceous (i.e., dwarf shrub) vegetation to indicate the overall severity of browning to the dominant ericoid vegetation in each plot. The chi‐square test, the standard measure of fit in SEM, was used as a primary indicator of whether model fit was acceptable. The Comparative Fit Index (CFI), Root Square Mean Error of Approximation (RMSEA) and Standardized Mean Square Error of Approximation (SRMR) were used to provide additional assessment of model fit.

## 3 RESULTS

### 3.1 Gross primary productivity

**Figure 1:**
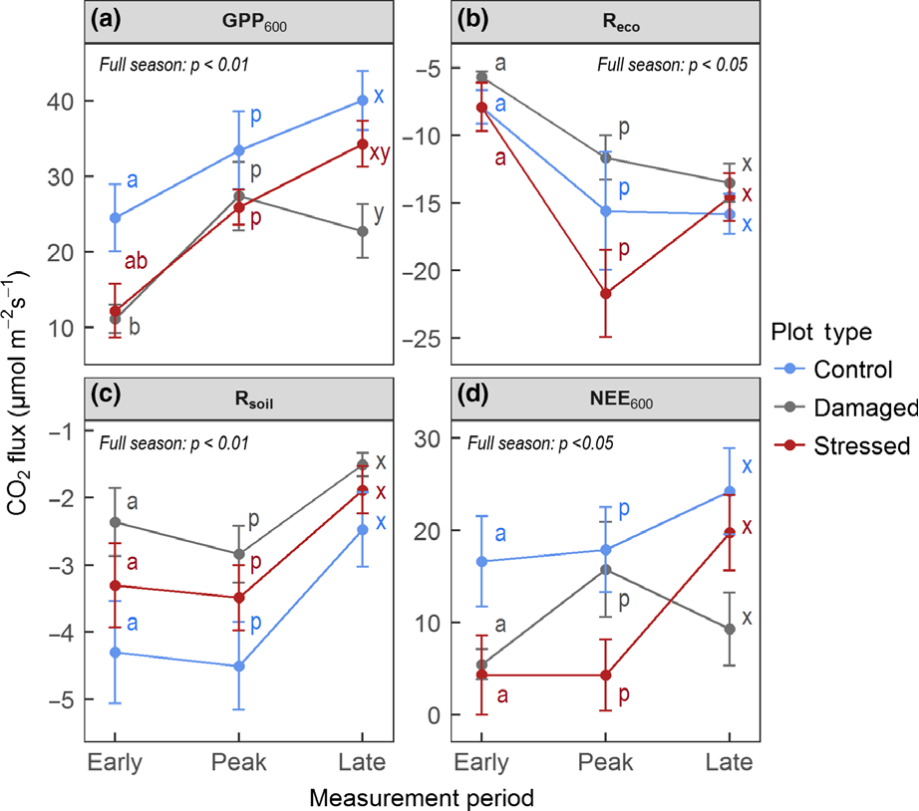
Change throughout the growing season in key ecosystem CO_2_ fluxes; (a) GPP_600_; (b) ecosystem respiration (dark CO_2_ release from soil and vegetation); (c) *R*_soil_ and (d) NEE_600_ in damaged (dominated by previous mortality, grey coloured), stressed (dominated by high anthocyanin pigmentation, dark red coloured) and control (green) plots. Positive values represent CO_2_ uptake (µmol m^−2^ s^−1^). Straight lines between data points are not intended to indicate a known linear transition between times of year, but are used to help link plot type across the year. Error bars represent one standard error. Letters represent significant differences as assessed by Tukey Honest Significant Differences; tests apply within measurement periods. “Full season” p values show differences between plot types across all time points. *N* = 16 within each measurement period

Across the growing season as a whole, mean GPP_600_ in both damaged and stressed plots was significantly reduced compared to controls by 37% and 23%, respectively (*F* = 8.52 *df *= 2,42, *p* < 0.001, Tukey HSD *p* < 0.05). Within early season, GPP_600_ was significantly affected by plot type (*F* = 5.19, *df* = 2,12, *p* < 0.05), with GPP_600_ in damaged plots reduced by an average of 55% (Tukey HSD *p* < 0.05, Figure 1a). At peak season, differences between plot types were not significant. By late season, GPP_600_ was significantly affected, with GPP_600_ in damaged plots reduced by an average of 43% (Tukey HSD *p* < 0.05).

### 3.2 Ecosystem and soil respiration

Across the season as a whole *R*_eco_ was significantly affected by plot type (Figure 1b; *F* = 3.44, *df* = 2,42, *p* < 0.05). *R*_eco_ was increased in stressed plots across the growing season compared to damaged plots (Tukey HSD *p* < 0.05), particularly at peak season, when *R*_eco_ in stressed plots was almost double that in damaged plots (though not statistically significantly so). Similarly, at peak season there was some evidence for elevated dark respiration in apical shoots from stressed plots (Supporting Information Section S2). In damaged plots and, most markedly, in stressed plots, there was a significant increase in *R*_eco_ from early to peak season (stressed plots: *F* = 7.565, *df* = 2,11, *p* < 0.01, damaged plots: *F* = 10.56, *df* = 2,15, *p* < 0.01), while in control plots *R*_eco_ did not change significantly between measurement periods.

*R*_soil_ was significantly affected by plot type across the season as a whole (Figure 1c; *F* = 8.116, *df* = 2,38, *p* < 0.01), with a mean reduction in damaged plots of 40% (Tukey HSD *p* < 0.01) and a mean reduction of a near significant 23% in stressed plots (Tukey HSD *p* = 0.057). Despite these differences being apparent across all three measurement periods combined, they were not statistically different in Tukey multiple comparisons within each time period (*p* > 0.05). On average across all plots, *R*_soil_ was constant between early and peak season, after which it declined by just under half by late season (*F* = 6.87, *df* = 2,43, *p* < 0.01).

### 3.3 Net ecosystem exchange

Differences in NEE_600_ between plot types (Figure 1d) were similar to those seen for GPP_600_. Across the full growing season, plot type had a significant effect on NEE_600_ (*F* = 4.718, *df* = 2,42, *p* < 0.05). Mean NEE_600_ overall was approximately halved in both stressed and damaged plots compared to controls (reductions of 50% and 48%, respectively, Tukey HSD *p* < 0.05). The effect of plot type on NEE_600_ was near significant within early season (*F* = 3.461, *df* = 2,12, *p* = 0.065), and within late season (*F* = 3.43, *df* = 2,12, *p* = 0.064), with early season showing the most substantial reductions in mean NEE_600_ in both damaged and stressed plots of 74% and 67%, respectively. Differences between plot types at peak season were not significant.

### 3.4 Shoot growth

**Figure 2:**
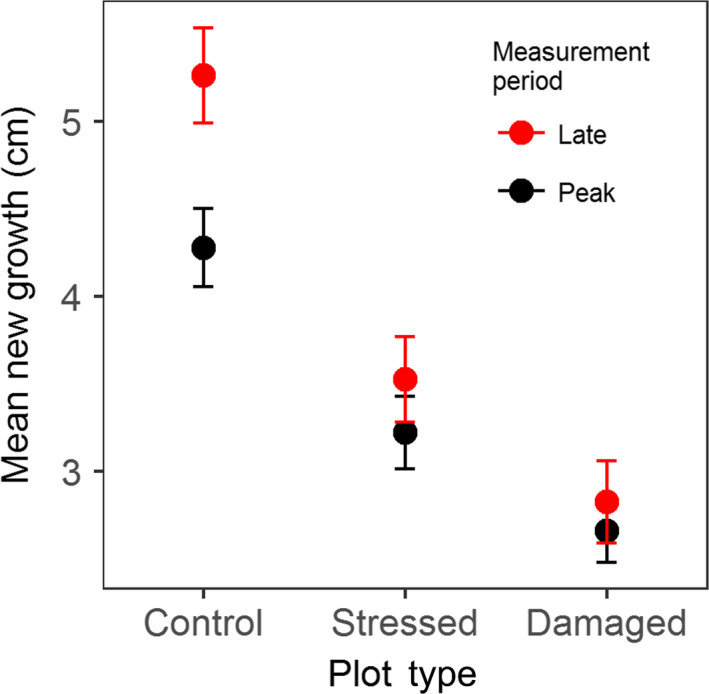
New (first‐year) growth (cm) at shoot apices within each plot type (control plots, stressed plots dominated by dark red anthocyanin pigmentation and damaged plots dominated by shoot mortality) at peak and at late season. Error bars represent one standard error. *N* = 57 at peak season, *N* = 55 at late season

The mean length of new shoot growth was significantly lower in both damaged plots and stressed plots compared to controls (38% and 27% lower, respectively) at peak season (Figure 2; *F* = 16.76, *df* = 2,54, *p* < 0.001, Tukey HSD *p* < 0.05). Growth reductions in both plot types compared to controls remained significant at late season (*F* = 24.89, *df* = 2,52, *p* < 0.001, Tukey HSD *p* < 0.05).

### 3.5 Decomposition

**Figure 3:**
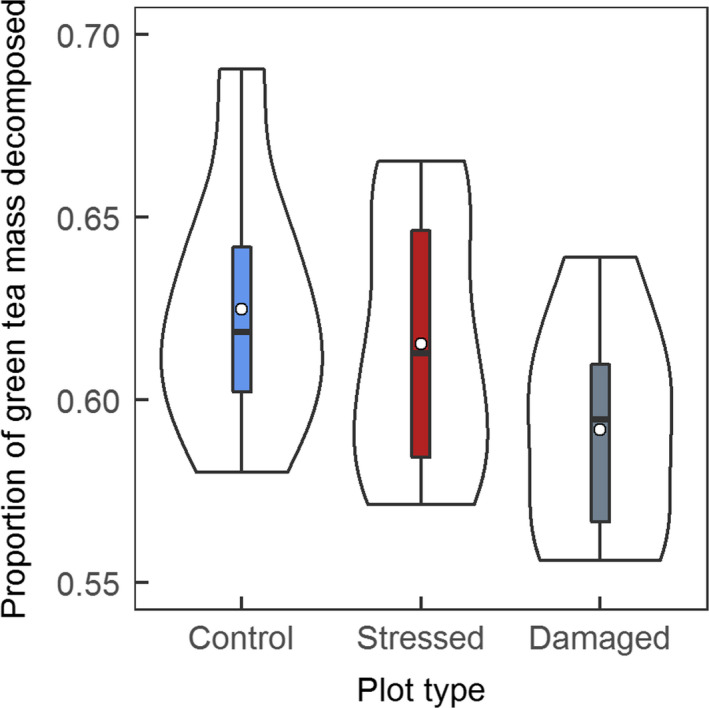
Proportion of green tea decomposed over 3 months in each plot type (control plots, stressed plots dominated by dark red anthocyanin pigmentation and damaged plots dominated by shoot mortality). Outer shape surrounding each box plot is a mirrored density plot showing data distribution. White points show means and black bars show medians. *N* = 29 [Colour figure can be viewed at wileyonlinelibrary.com]

No significant differences in fraction decomposed of rooibos tea were found between plot types. However, there was a near significant difference in fraction decomposed of green tea between plots, with lower decomposition seen in damaged plots compared to control plots (Figure 3, *F* = 3.313. *df* = 2,26, *p* = 0.052, Tukey HSD *p* = 0.053). This difference corresponds to a near significant difference in litter stabilization, which was higher in damaged plots when compared to controls (*F* = 3.313. *df* = 2,26, *p* = 0.052, Tukey HSD *p* = 0.053).

### 3.6 Change in anthocyanin pigmentation

**Figure 4:**
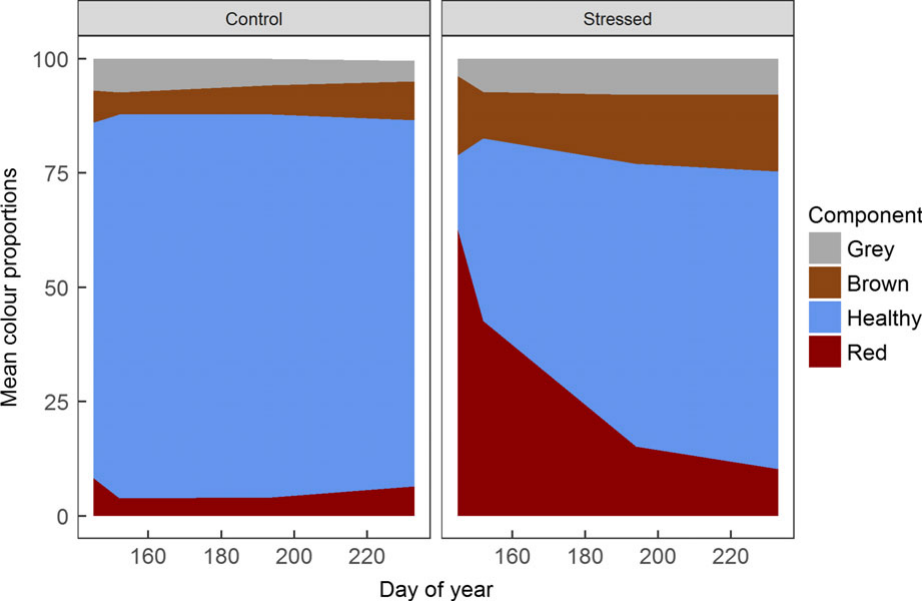
Mean proportions of total leaf area of tagged apical shoots exhibiting different states, as reflected by leaf colour. Grey indicates leaf mortality from a previous growing season (followed by degradation of leaf pigment over winter), brown indicates fresh mortality, red indicates high anthocyanin pigmentation (signalling stress), and healthy green leaf area is shown here in blue. *N* = 20

Tagged shoot data (Figure 4) showed high levels of anthocyanin pigmentation (dark red coloration) at early season in shoots located in stressed plots (mean of 63%) compared to those located in control plots (mean of 8%). Dark red anthocyanin pigmentation in shoots located in stressed plots fell significantly (*p* < 0.05) within the early season measurement period and from early to peak season. A slight, nonsignificant further decline was seen by late season. In control plots, levels of dark red pigmentation did not change significantly, but were highest at the start of the growing season and lowest at peak season. Proportions of grey (past mortality) and brown (fresh mortality) coloration did not change significantly throughout the season in either shoots located in control plots or stressed plots. However, there was some increase in the proportion of brown coloration in both plot types; an increase of 7% from June to September in stressed plots and of 4% in control plots.

### 3.7 Relationships between CO_2_ fluxes and browning severity across plots

**Figure 5:**
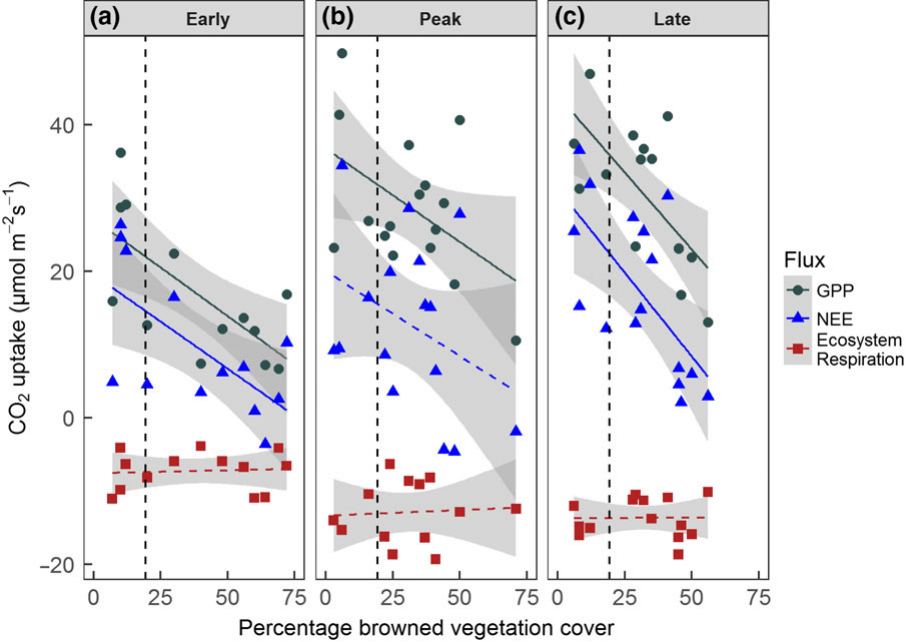
Correlations between total browned vegetation cover (including previous and fresh mortality and anthocyanin pigmentation stress response) and key ecosystem CO_2_ fluxes GPP_600_ (grey), NEE_600_ (blue) and *R*_eco_ (red) during (a) early, (b) peak, and (c) late season. Positive values represent CO_2_ uptake. Solid lines indicate statistically significant correlations; dashed lines indicate correlations where *p* > 0.05. While these data represent CO_2 _flux measurements repeated at the same plots across the growing season, cover of damaged vegetation changes throughout the season due to recovery and change in cover of herbaceous species. Thus, the percentage cover of browning in each plot varies throughout the season. *N* = 16 for each flux within each measurement period [Colour figure can be viewed at wileyonlinelibrary.com]

Negative correlations between GPP_600_ and total cover of browned (stress and damage combined) vegetation across flux plots were present throughout the growing season (Figure 5). These correlations were highly significant at early and late season (early: *F* = 13.41, *df* = 1,13, *p* < 0.01, late: *F* = 15.88, *df* = 1,14, *p* < 0.01) and near significant at peak season (*F* = 4.312, *df* = 1.14, *p* = 0.057).

Correlations between GPP_600 _and total browning correspond to substantial reductions in productivity at mean cover of total browning across the site, as assessed by transect surveys (Supporting Information Section S1). During early season, GPP_600_ at mean site‐level browning was reduced by an estimated 34% compared to that in undamaged vegetation, with a 15% reduction seen at peak season and a 20% reduction at late season. Multiple regression using separate terms for past damage (grey shoot mortality) and recent browning (brown fresh shoot mortality and dark red pigmentation) was also significant at early and late season, but did not provide significantly improved model fit compared to linear regression.

*R*_eco_ did not correlate with total browned cover at any point during the season (Figure 5). *R*_soil_ declined with increasing area of total browned vegetation throughout the growing season (Supporting Information Figure S4). This correlation was significant at early season (*F* = 6.02, *df* = 1,11, *p* < 0.05) and near significant at peak (*F* = 4.12, *df* = 1,14, *p* = 0.062) and late (*F* = 4.16, *df* = 1,12, *p* = 0.064) season. *R*_soil_ at mean cover of total browning was reduced by between 26% at early season and 16% at peak season. Multiple correlations using separate terms for past damage and recent browning were not significant.

NEE_600_ was negatively correlated with total cover of browned vegetation (including mortality and stress) during early (Figure 5a; *F* = 10.34, *df* = 1,13, *p* < 0.01) and late season (Figure 5c; *F* = 10.76, *df* = 1,14, *p* = 0.01), but not during peak season (Figure 5b). These correlations corresponded to substantial estimated site‐level NEE_600_ reductions when combined with mean cover of total browning across the site (Supporting Information Section S1), from a reduction of 44% compared to healthy vegetation during early season to 28% during late season. Multiple correlations using separate terms for past damage and recent browning were also significant at early and late season, resulting in higher estimated NEE_600_ reductions (reaching 52% at early season), and were marginally significant at peak season, providing an estimated site‐level NEE_600_ reduction of 29%.

### 3.8 Structural equation modelling

**Figure 6:**
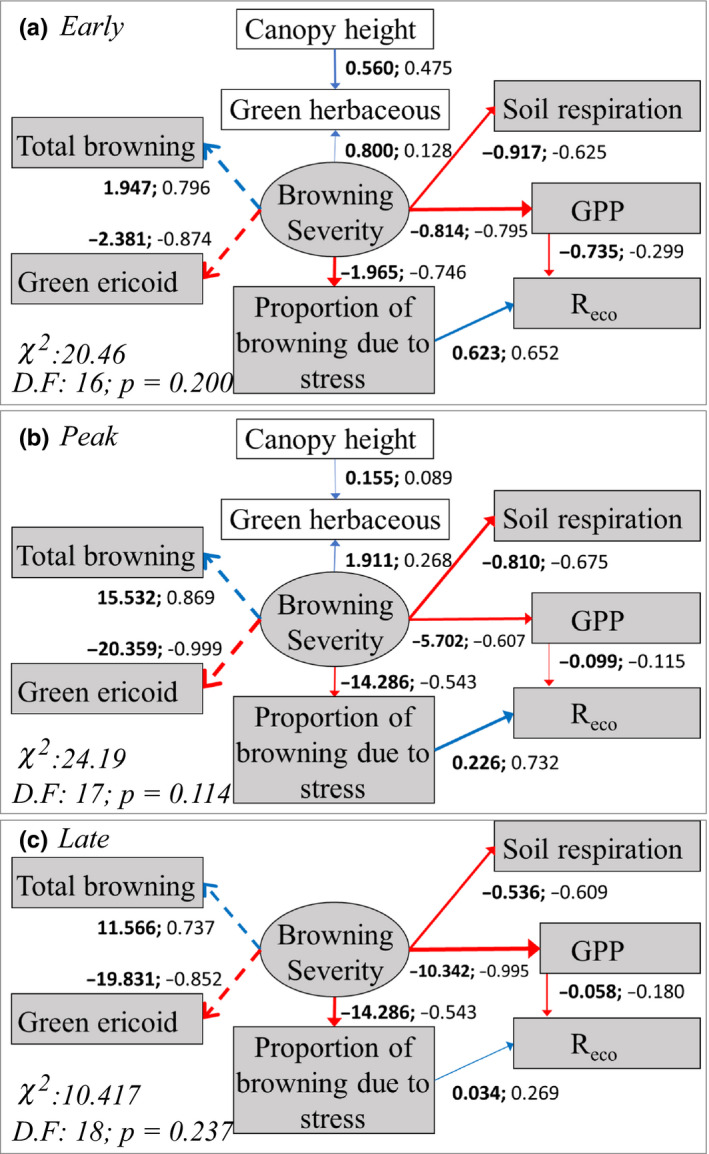
Structural Equation Models of early (top), peak (centre) and late (bottom) season data, showing the relationships between “Browning severity” (a latent variable representing the impact of extreme conditions on the cover of the dominant ericoid vegetation) and CO_2_ fluxes (GPP_600,_ *R*_eco_ and *R*_soil_). Core model is shown in grey with modifications in white (see methods). Measured variables are shown as squares, latent variables as circles, and paths between indicator variables and the latent variable they describe as dashed lines. Red lines indicate negative and blue lines positive effects, with annotations showing unstandardized (bold) and standardized coefficients. During late season, modification of the established core model with cover of herbaceous species and canopy height was rejected (*p* < 0.001). Statistically significant model fit in SEM is indicated by *p* > 0.05

Structural equation modelling supported the above analyses. “Browning Severity,” a latent, unmeasured variable (see methods) combining percentage cover of total browned vegetation (positive effect on Browning Severity) and remaining green ericaceous vegetation (negative effect on Browning Severity), had a strong, negative impact on both GPP_600_ and *R*_soil_ throughout the growing season (Figure 6). As conventional analyses found no site‐level relationship between total browning and *R*_eco_, this relationship was not included in SEM. However, since comparisons between plot types indicated a difference in the response of *R*_eco_ to browning associated with past mortality (grey coloured) compared with stress (dark red), an indirect relationship was modelled between Browning Severity and *R*_eco_ via the proportion of total browning associated with stress (as measured during early season). This showed that until late season (a) the proportion of browning initially associated with stress had a highly significant, positive relationship with *R*_eco_ (in agreement with comparisons between plot types, Figure 1c); (b) the proportion of browning associated with stress had a highly significant, negative relationship with Browning Severity; meaning the most heavily damaged plots were dominated by past mortality (grey coloured shoots), rather than recent stress (dark red anthocyanin pigmentation); and (c) there was a significant, negative indirect relationship between Browning Severity and *R*_eco; _meaning that when a high proportion of browning was associated with past mortality, *R*_eco_ was reduced. This indirect relationship was not significant at late season.

Conventional analysis also showed that damaged plots contained significantly greater cover of herbaceous species compared to controls (data not shown). It was hypothesized that this greater cover of herbaceous species with high productivity in more heavily damaged ericaceous vegetation may contribute to the weaker overall GPP_600_‐browning correlation seen at peak season (following leaf‐out of herbaceous species), compared to early and late season. Greater herbaceous cover could result from either altered conditions (e.g., higher soil moisture or reduced competition for space, light or nutrients due to reduced cover of dominant evergreen dwarf shrubs) following browning, or from the established tendency for taller vegetation both to facilitate co‐occurring species, for example, by reducing wind speeds and temperature variability during spring, and simultaneously to be more vulnerable to the effects of winter extreme events (Bjerke et al., 2014; Brooks et al., 2011; Goetz, Bunn, Fiske, & Houghton, 2005). This distinction is significant; the latter implies a process arising from patch‐scale heterogeneity which may reduce the mean impact of browning on productivity, but is likely to vary in importance with vegetation structure and to operate at small scales, while the former implies a broader scale mechanism arising directly from browning through which reductions in productivity following browning may be slightly, but consistently, ameliorated. These ideas were explored via modification of the core SEM through the addition of a variable for green herbaceous cover and of paths between this new variable and both Browning Severity and canopy height (representing regressions on green herbaceous cover). The modified SEM was an acceptable fit at early and peak season and indicated a near significant (*p* = 0.052) positive effect of canopy height on herbaceous cover at early season. However, there was no significant correlation between Browning Severity and cover of green herbaceous species.

## 4 DISCUSSION

This detailed assessment of the impacts of Arctic browning driven by extreme climatic events on ecosystem CO_2_ fluxes has shown that these events can considerably reduce gross and net ecosystem CO_2_ uptake, with substantial consequences for soil processes and carbon sink capacity. Furthermore, the magnitude of impact was similar between mortality and stress responses, such that both need to be considered when quanitfying the impacts of extreme event‐driven browning on Arctic ecosystem carbon balance.

### 4.1 Gross primary productivity and growth

Mean GPP_600_ (Gross Primary Productivity standardized at 600 µmol m^−2^ s^−1^; a moderate light level) was significantly lower in damaged and stressed plots across the growing season as a whole compared to control plots, in accordance with hypothesis (i).

When measurement periods are considered individually, GPP_600_ in damaged plots was lower compared to control plots at early and late season, but not at peak season. This is contrary to hypothesis (ii), that reductions in CO_2_ fluxes associated with browning would diminish throughout the growing season. Instead, this suggests that the greatest impacts on ecosystem CO_2_ uptake may occur towards the shoulder seasons. The lack of significant differences between plot types at peak season is likely due to a combination of factors including resprouting and recovery of damaged vegetation, and leaf‐out of highly productive herbaceous species. Browned plots contained significantly more herbaceous cover compared to controls, meaning that leaf‐out of these species will have had a larger positive impact on productivity in these plots. Structural Equation Modelling (SEM) indicated that this difference in herbaceous cover between plot types is linked to canopy height (Figure 6); previous work has shown that taller vegetation is more severely damaged following winter extreme events (Bjerke et al., 2017), while SEM showed that during early season taller vegetation also supported more herbaceous species (including graminoids); the latter relationship was also present when analysing control plots alone (data not shown). This is likely due to the facilitative effects of a taller canopy, such as reducing spring wind speeds (Brooks et al., 2011; Goetz et al., 2005; Sturm et al., 2005). Thus, higher herbaceous cover in damaged plots is probably not a result of browning, but rather a pre‐existing co‐variate of canopy height. This could indicate a mechanism providing some mitigation of the impact of browning on GPP within a landscape.

Although reductions in GPP_600_ within measurement periods were statistically significant only where driven by mortality, mean GPP_600_ reductions associated with stress (though not statistically significant) were of a similar magnitude to those driven by mortality at early and peak season (Figure 1a). While previous work has shown anthocyanin production can reduce photosynthetic capacity (Burger & Edwards, 1996; Steyn et al., 2002), it is nonetheless surprising that stress might result in such considerable reductions in plot‐level CO_2_ uptake. Such reductions in productivity are reflected in first‐year growth of live shoots, which in accordance with hypothesis (iii) was reduced in stressed and damaged plots (Figure 2), possibly due to delayed bud burst (Bokhorst et al., 2008), transient physiological damage (Bokhorst et al., 2010) or the metabolic cost of anthocyanin synthesis (Zangerl, Arntz, & Berenbaum, 1997).

### 4.2 Soil and ecosystem respiration

Overall, *R*_soil_ (dark CO_2_ release from soil) was reduced in damaged and—with near significance (*p* = 0.057)—in stressed plots across the three measurement periods (Figure 1c). This supports previous work showing that vegetation damage following climatic and biological extreme events reduces *R*_soil_ (Moore et al., 2013; Olsson, Heliasz, Jin, & Eklundh, 2017; Zhao, Peichl, & Nilsson, 2017), and further demonstrates that vegetation stress may have qualitatively similar effects to damage (albeit of a lesser magnitude). Reductions in *R*_soil_ following vegetation damage have previously been attributed to diminished microbial activity and deceleration of soil processes due to reduced transfer of carbon below ground (Litton, Raich, & Ryan, 2007; Parker et al., 2017). Here, decomposition data are consistent with this mechanism, given that decomposed fraction of green tea (labile litter) was lower in damaged plots, indicating reduced microbial activity. For soil respiration measurements, soil collars were installed 3–5 days prior to first measuring soil respiration; while this should be sufficient for larger, initial disturbance effects (e.g., from cut roots) to stabilize, soil respiration measurements could still be influenced by these effects (resulting in some overestimation in all plots). While it is not unusual for flux measurements to be taken a few days (or even hours) after collar insertion, ideally collars should be inserted during the growing season before.

The respective impacts of vegetation stress and damage on *R*_eco_ (dark ecosystem CO_2_ release, including from soils and vegetation) are less consistent. In damaged plots, *R*_eco_ was slightly, but not significantly, lower than in controls. However, these plots were exposed to an extreme event 2 years prior to measurements (in contrast to stressed plots, in which exposure occurred in the winter prior to measurements), possibly affecting measured respiration responses. Two years of vegetation recovery may increase respiration of plants (presence of more metabolically active tissue) and soil (greater below‐ground carbon allocation), and measured reductions in respiration might therefore be an underestimate of what they were immediately following damage. In stressed plots, *R*_eco_ was increased across the growing season compared to damaged plots (Figure 1b). Reduced soil respiration implies that this increase in *R*_eco_ was driven by increased respiration of vegetation, an effect which SEM analysis indicates was greatest at peak season. That this increased vegetation respiration is directly linked to stress, as indicated by the high anthocyanin pigmentation observed in *C. vulgaris*, is supported by shoot‐level measurements showing a marginally significant increase in dark respiration of visibly pigmented shoots compared to green shoots (Supporting Information Section S2). However, we cannot be certain whether that link between increased pigmentation and respiration is causal (e.g., greater respiration from the metabolic cost of anthocyanin production) or simply correlative (pigmentation and respiration increases are both stress responses, but one does not drive the other). Nonetheless, stress associated with exposure to winter extreme events appears to increase respiration and likely reflects greater maintenance and repair needs, as documented following drought, herbivory and mechanical damage (Flexas, Galmes, Ribas‐Carbo, & Medrano, 2005; Strauss, Rudgers, Lau, & Irwin, 2002; Zangerl et al., 1997).

### 4.3 Net ecosystem exchange

Major impacts on NEE_600_ (Net Ecosystem CO_2_ Exchange standardized at 600 µmol m^−2^ s^−1^; a moderate light level), which varied substantially across the growing season, were seen in both stressed and damaged plots (Figure 1d). Mean NEE_600 _was reduced by 48% and 50% across the growing season in damaged and stressed plots, respectively, compared to controls. NEE_600_ is the primary measure of ecosystem carbon balance; this therefore represents a substantial impact on sequestration capacity, particularly in an ecosystem with a long winter where the annual carbon sequestration therefore depends on a short, dynamic growing season (Larsen et al., 2007).

Differences in NEE_600_ between plot types at individual measurement periods were not statistically significant. However, the magnitude of differences in mean NEE_600_ between stressed and damaged plots at peak season, combined with contrasting impacts of stress and damage on *R*_eco_, indicates potentially important differences in how stress and damage affect NEE_600_. Specifically, while mean NEE_600_ in damaged plots increased substantially between early and peak season, mean NEE_600_ in stressed plots did not (due to the rapid increase in respiration of stressed vegetation discussed above), and thus, NEE_600_ was 73% lower in stressed plots than in damaged plots at peak season. However, although NEE_600_ did not increase in stressed plots between early and peak season, a substantial proportion of vegetation in these plots had “re‐greened” following breakdown of anthocyanin pigments during the same period. Future work should therefore consider this potential difference in peak season impacts of stress and damage on NEE_600_, and the possibility that where substantial stress follows extreme event exposure, impacts on NEE_600_ may not be predictably related to loss of greenness at peak season, and may therefore be harder to detect.

### 4.4 Change in anthocyanin pigmentation

In shoots from stressed plots, the mean proportion of leaf area exhibiting stress (dark red anthocyanin pigmentation) was more than 40% in early June (DOY ~ 155), and more than 15% at peak season (DOY ~ 195; Figure 4). This is markedly higher compared to both control shoots (mean 4% dark red in June), and to previous observations of spring pigmentation in evergreen dwarf shrubs, which typically falls to its lowest levels by early‐ to mid‐June and/or shortly after snow melt (Mac Arthur & Malthus, 2012; Oberbauer & Starr, 2002). This demonstrates that this response is beyond usual levels of pigmentation following snow melt. While pigmentation was much more persistent than that seen naturally after snow melt, it did none‐the‐less diminish rapidly from the very high levels seen early in the season, dropping from 63% in May (DOY ~ 145) to 43% in June (DOY ~ 155). This aligns with recovery of the dwarf shrub *Cassiope tetragona* on Svalbard during summer 2015 (Anderson et al., 2016) from frost injury caused by lack of sufficient winter snow cover (Callaghan, Carlsson, & Tyler, 1989).

While the rapid decline in the proportion of dark red leaf area observed in our work largely reflects breakdown of anthocyanin pigments and a resulting increase in green leaf area, there is also evidence of mortality occurring in highly pigmented leaves (resulting in conversion from dark red leaf area to brown). This suggests that while highly pigmented *C. vulgaris* shoots were often able to “re‐green,” they also experienced increased leaf mortality across the season compared to unaffected shoots. While anthocyanins are known to be synthesized to provide plant protection (Gould, 2004; Landi et al., 2015), the possibility that persistent pigmentation may signal plant damage and be followed by elevated mortality is little understood.

### 4.5 Browning: CO_2_ flux relationships across plots

The extreme conditions during winter 2013/14 which caused mortality at this site resulted in similar damage across the Lofoten region, and even other regions of sub‐Arctic and boreal Norway as much as ~600 km south and ~700 km east of our study site (Bjerke et al., 2017; Meisingset, Austrheim, Solberg, Brekkum, & Lande, 2015), indicating that these vegetation responses are increasingly relevant even at regional or greater scales. Therefore, simplified relationships between browning and CO_2_ flux would be beneficial for upscaling these impacts.

GPP_600_ was negatively correlated with percentage cover of total browned vegetation throughout the growing season, demonstrating for the first time that the extent of visible browning was clearly and consistently related to the impact on plot‐level gross CO_2_ uptake, regardless of whether browning was primarily a result of mortality or stress (Figure 5) (though see caveats below when attempting to apply these relationships to NEE). When scaled to the site level, these linear negative correlations of GPP_600_ to browning translated to substantial reductions in GPP_600_ at site‐level of up to 34%, demonstrating the considerable capacity of browning events to reduce carbon sequestration even at larger scales where there may be a mosaic of damaged and undamaged vegetation. The emergent relationships also support hypothesis (iv) that CO_2_ fluxes would correlate negatively with overall browning. It also adds to previous work identifying emergent relationships between leaf area index and CO_2_ fluxes that act across contrasting vegetation types and help simplify the heterogeneity of vegetation distribution and productivity in Arctic regions (Street et al., 2007, Shaver *et al.,* 2007). That work can greatly facilitate regional estimates of carbon balance and its response to climate change, and we suggest that for GPP, such relationships can now also include vegetation damaged by extreme events.

*R*_soil_ was also negatively correlated with total browning, further reflecting the slowdown in below‐ground processes which follows a reduction in aboveground productivity (Supporting Information Figure S3; Litton et al., 2007; Parker et al., 2017). However, contrary to hypothesis (iv), no overall correlation between total browning and *R*_eco_ was identified. Increased *R*_eco_ in stressed plots compared to damaged plots suggests this is due to opposing impacts on vegetation respiration of mortality and stress, which appear to decrease and increase vegetation respiration, respectively. This is further supported by SEM, which indicated that browning severity had a negative impact on *R*_eco_ when the influence of vegetation stress was controlled for.

As was seen with GPP_600_, NEE_600_ correlated linearly and negatively with total browning (including both damaged and stressed vegetation) at early and late season, translating to site‐level reductions of between 25% and 44% across the growing season (Figure 5), and demonstrating the substantial capacity for browning events to reduce site‐level carbon sink strength. However, at peak season there was no correlation between NEE_600_ and total browning, due to the contrasting impacts of damage and stress on vegetation respiration. Interestingly, a multiple regression of peak season NEE_600_ including the two terms “past damage (grey mortality) recorded at *peak* season” and “stress and fresh damage (brown mortality) recorded at *early* season” provided a significant correlation. The fact that this links the stress response seen at early season to peak season NEE_600_ is consistent with the idea of a legacy effect of early season stress, such that this has a continued respiration cost into peak season—even though re‐greening of stressed tissues means peak season measures of visible browning no longer correlate with peak season NEE_600_. This needs further investigation, but may be an important caveat to using simplified relationships that predict NEE from leaf area index or greenness indices such as NDVI (Street et al., 2007, Shaver *et al.,* 2007); where winter extreme events cause substantial vegetation stress, assessing their impacts on NEE according to peak season greenness alone could substantially underestimate the reduction in net CO_2_ uptake capacity—by almost 10% in this case. However, note our conclusion earlier that these predictive relationships still hold true for GPP.

Overall, this work shows that both stress and mortality following exposure to winter climatic extreme events can have major impacts on plant physiology and ecosystem function, including significantly reducing ecosystem CO_2_ uptake (hypotheses i and iii). While many of these impacts are present across the growing season, mean reductions in GPP were most substantial early or late in the season (hypothesis ii). This highlights the need to support peak season measurements with full‐season assessments and adds to an increased appreciation of the role of shoulder seasons in Arctic biogeochemical cycling (Grogan & Jonasson, 2005, 2006; Oechel, Vourlitis, & Hastings, 1997; Zona et al., 2016). Importantly, over a single growing season, stress indicated by dark red pigmentation was just as important for reducing CO_2_ uptake as mortality and continued to reduce CO_2_ sequestration (due to ongoing greater respiration) following recovery of greenness (hypothesis iv). This adds an additional challenge to detecting and upscaling the impacts of vegetation stress on CO_2_ uptake on a regional or global scale using remote sensing (Park et al., 2016). Both extensive mortality and visible stress are common responses following exposure to extreme events such as frost drought, icing and extreme winter warming, which are predicted to increase in frequency with climate change (Johansson et al., 2011; Vikhamar‐Schuler et al., 2016). The scale of the impacts of mortality and stress on ecosystem CO_2_ fluxes across the growing season reported here highlight a clear need for a process‐based understanding of the impacts of such events on Arctic vegetation and CO_2_ balance. This understanding is central to predicting both how vegetation will respond to continuing rapid climate change at high latitudes, and how these responses may feedback to climate.

## AUTHOR CONTRIBUTIONS

Project conceived and designed by RT, GKP, JWB and HT. Fieldwork designed and data acquired by RT and LS, with logistical and scientific support from GKP, JWB and HT. Data analysis carried out by RT with support from GKP, JWB and HT. Manuscript preparation led by RT, with support (substantial critical feedback, revisions and additions to text) from all co‐authors. Final version of the manuscript read and approved by all co‐authors.
