# Supplementary material for: Arctic browning: Impacts of extreme climatic events on heathland ecosystem CO2 fluxes
Source: Glob Chang Biol. 2018 Nov 25;25(2):489–503. doi: 10.1111/gcb.14500 (PMC7379734; doi:10.1111/gcb.14500)
Supplement: Supplementary file 1 [file GCB-25-489-s001.docx]

**Figure S1**


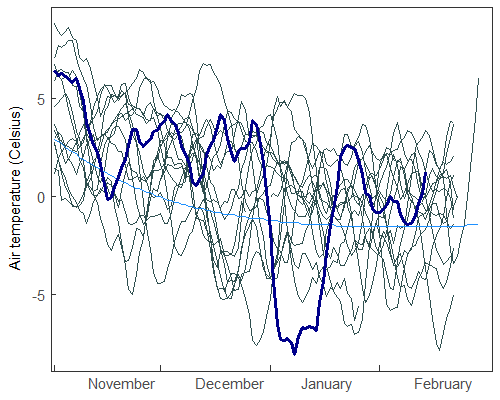


Fig. S1: Seven day moving average air temperature (˚C) values from November 1^st^ to February 29^th^. Each line shows values for one year; the 2015/16 winter is shown in dark blue, and the preceding 14 years in dark grey. Daily normal values are shown in light blue. Data obtained from the eklima.no database. While the 2015/16 winter temperatures are not outside those seen in other years, it is the combination of a period of warmth which melted snow and exposed plants, followed by a large and rapid temperature drop, that resulted in the frost-drought damage in that winter,

**Figure S2**


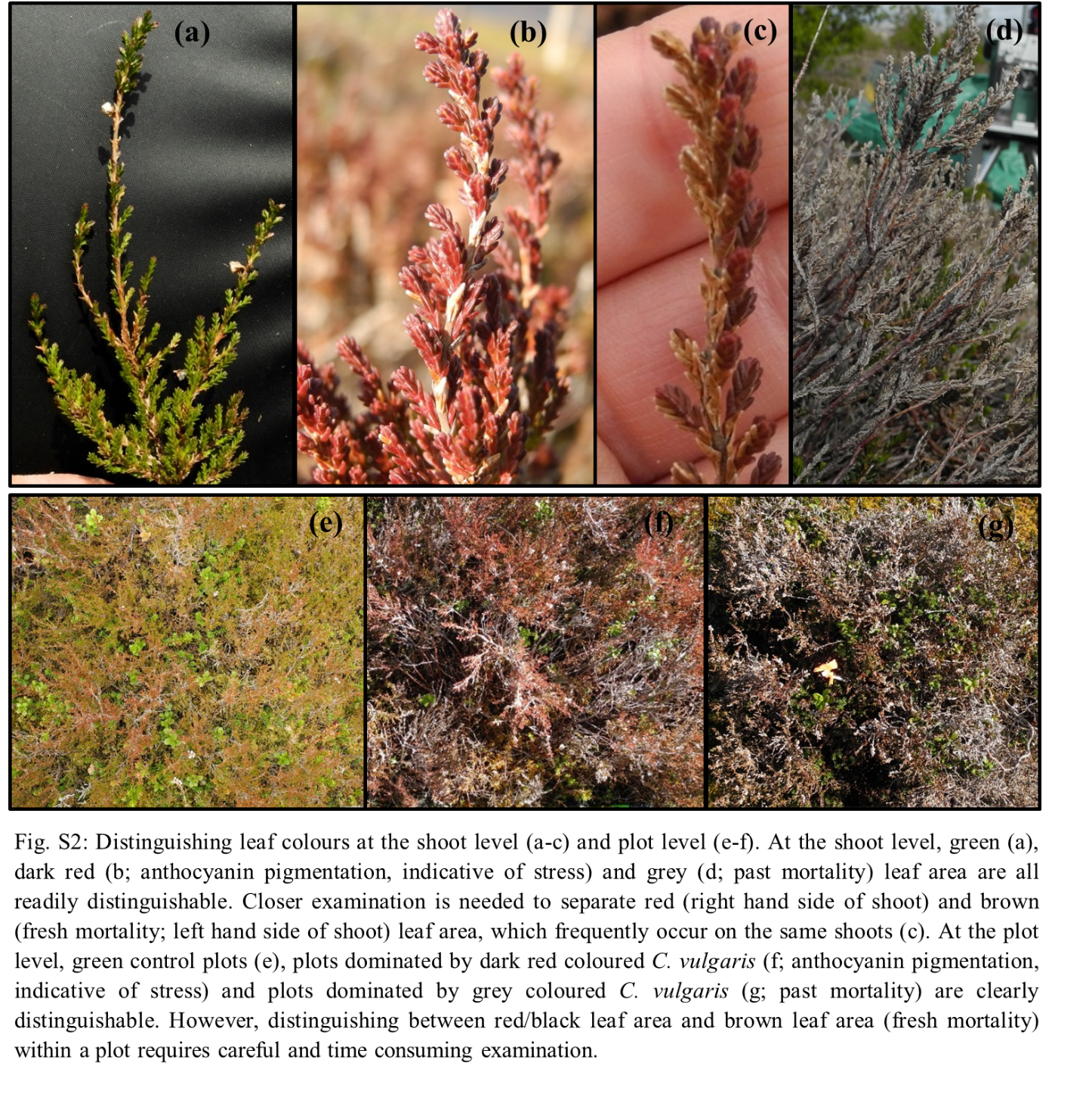


**Figure S3**

**
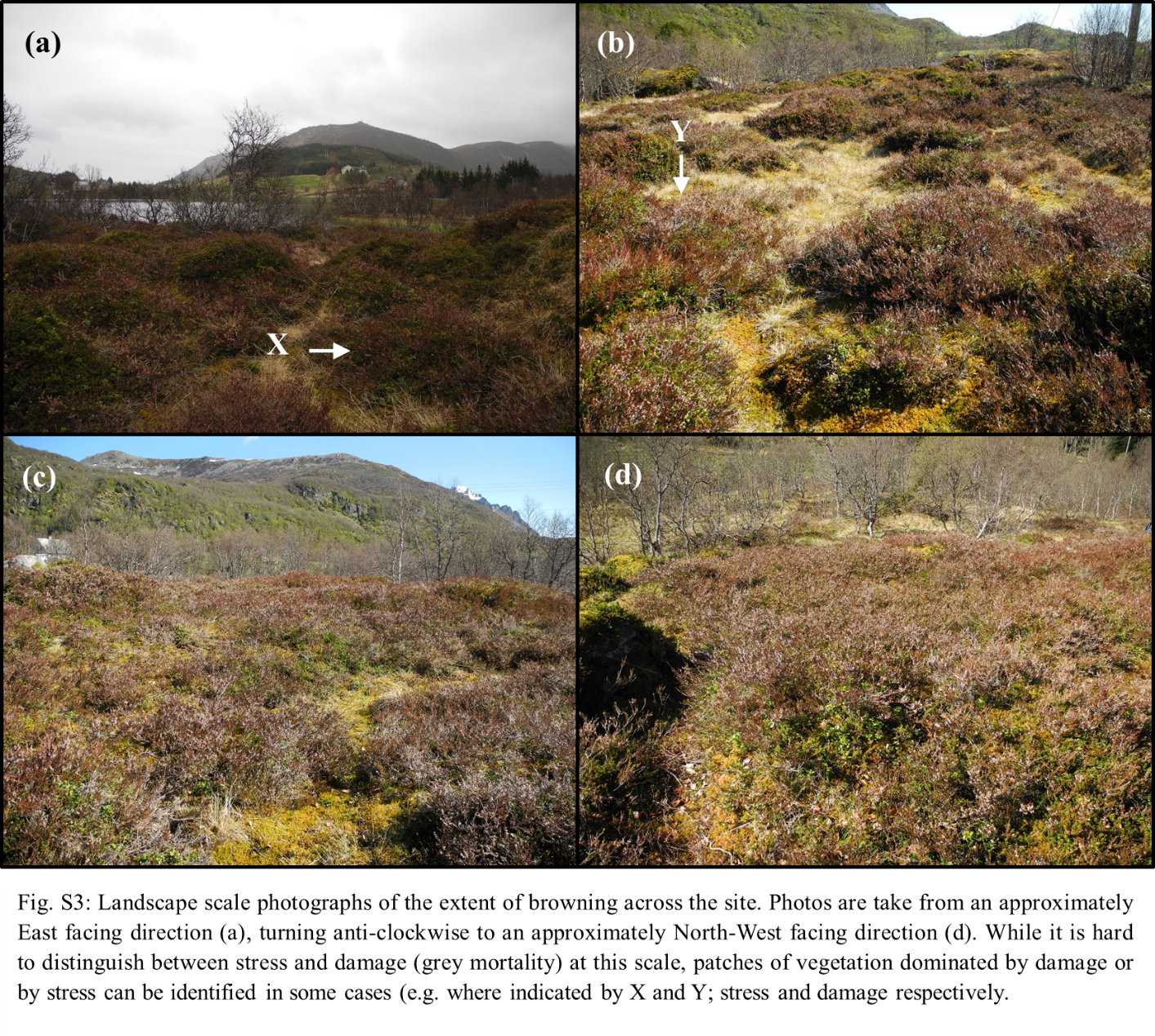
**

**Table S1**

Table. S1: Mean percentage cover of different categories of vegetation types at the site-level, as measured by transect surveys, and across all plots.

**Section S1**

*Assessment of site-level browning*

Methods

To estimate site-level browning (including both stress and damage), two 15-18m transects were completed during each measurement period with percentage cover recorded in a 1m x 1m area using four 50 x 50cm quadrats at intervals of three metres. In each quadrat damage was assessed visually, along with percentage cover of dominant species, by recording percentage cover of green (healthy), grey (shoot mortality from which all pigmentation has leached, indicating damage is from a previous year), brown (fresh shoot mortality) and red/black vegetation (high anthocyanin pigmentation, indicating stress). While all three categories of browning (grey, brown, red/black) are readily distinguishable by eye at a shoot level (Fig. S2) close examination can be required to distinguish between fresh shoot mortality (brown) and anthocyanin pigmentation (red/black) at the plot level. The time consuming nature of the process of distinguishing between percentage cover of brown (fresh mortality) and red/black (stress) leaf area at the plot-level meant that these two damage types were recorded in combination (i.e. total damage) during transect surveys.

Results

Site transects showed substantial total browning (previous and fresh shoot mortality and high anthocyanin pigmentation combined) during the early season measurement period, with >50% cover of browning recorded at a quarter of plots. Significant browning remained visible by late season (Fig. S4). Within total browning, mean cover of fresh shoot mortality and high anthocyanin pigmentation combined (brown and red/black respectively) was 15.2% during early season. This fell significantly to 10.3% between early and peak season (t=3.0773, d.f.=63.913, p<0.01), before a further slight decrease to 9.5% by late season. Mean cover of previous shoot mortality (grey standing dead shoots from 2013-14 winter frost drought) was 22.2% during early season, falling significantly to 11.7% by peak season (t=5.5789, d.f.=78.486, p<0.001) with a further non-significant decrease by late season, likely due to fresh growth overtopping and obscuring dead material. Mean cover of apparently healthy, green vegetation increased from 70.3% during early season to 106.4% by mid-season, partly accounted for by a significant 15.2% increase in mean cover of herbaceous species (t=-6.1303, d.f.=111.806, p<0.001).


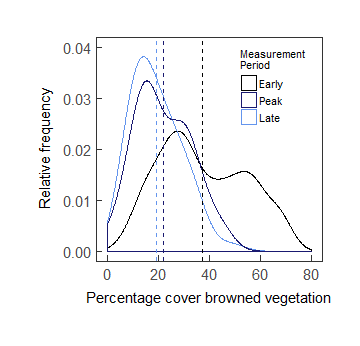


Fig. S4: Frequency polygon showing seasonal change in total percentage cover of all types of browned vegetation combined (including previous and fresh shoot mortality and anthocyanin pigmentation stress response), as recorded by transect surveys

**Figure S5**


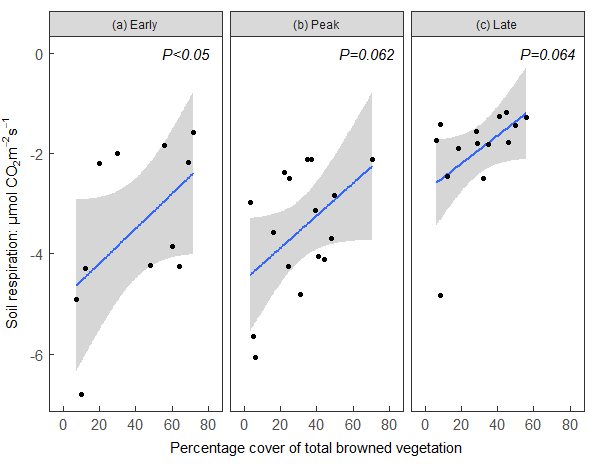


Fig. S5: Correlations between total browned vegetation cover (including previous and fresh mortality and anthocyanin pigmentation stress response) and soil respiration during early, peak and late season. Negative values represent CO_2_ release. While these data represent respiration measurements repeated adjacent to the same plots across the growing season, cover of damaged vegetation within those plots changes throughout the season due to recovery and change in cover of herbaceous species. Thus the percentage cover of browning in each plot varies throughout the season.

**Section S2**

*Shoot level CO_2_ exchange*

Methods

During the peak season measurement period, shoot level CO_2_ exchange was measured on shoots adjacent to and showing a similar level of anthocyanin colouration to tagged shoots (see ‘*assessment of change in anthocyanin pigmentation’* in the main text) in 5 control plots and 5 stressed plots, using an LI-6400 system connected to a LI-6400-05 transparent conifer chamber. PAR was recorded using a LiCor Quantum sensor mounted on an acrylic shelf outside the conifer chamber. Shoot CO_2_ exchange was recorded at 5 light levels, beginning with full light and adding three successive levels of shading, achieved using optically neutral shade cloths, before ending in darkness achieved using a tarpaulin. Shoot photosynthetic rate (µmol CO_2_ m^-2^ s^-1^) was allowed to stabilise at each light level (indicated by a coefficient of variation of photosynthetic rate of <0.1). Photosynthetic rate was converted to net CO_2_ flux per unit leaf area and modelled as a rectangular hyperbola with a term for respiration. Subtraction of the latter from CO_2_ flux enabled a light response curve of GPP to be fitted, and thus GPP to be standardised at a PAR of 600µmols PPFD m^-2^ s^-1^ (GPP_600_).

After measurements were complete, the extent of the shoot sealed within the conifer chamber was marked and clipped. Clipped shoot sections were stored in high humidity conditions provided by damp kitchen roll in polythene bags for 5 days to prevent drying and shrinkage, after which leaves and stems were separated. The leaves from each shoot were then scanned on a flatbed scanner and the resulting image analysed in Image J (Schneider *et al*., 2012) to obtain total leaf area for each shoot level measurement.

Results

There were no significant differences in photosynthetic activity between shoots from stressed and control plots (Fig. S6a). Shoots from stressed plots on average respired at a higher rate compared to those from control plots (Fig. S6b). Although this difference is not statistically significant (t=1.96, d.f.=5.83, p=0.099), it may nonetheless indicate elevated respiration in stressed shoots, particularly given the small sample size of this supporting dataset (n=9).

Fig. S6: (a) Mean Gross Primary Productivity and (b) mean dark respiration at 600 µmol PPFD m^-2^ s^-1^ in *C. vulgaris* shoots from green control plots and from stressed plots dominated by red/black anthocyanin pigmentation.


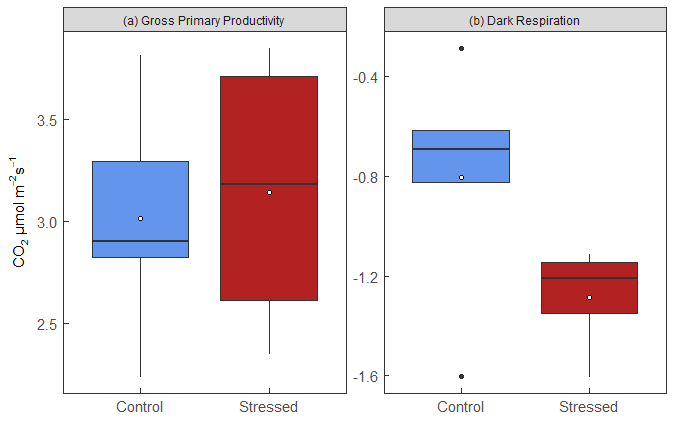


**References**

Schneider CA, Rasband WS, & Eliceiri KW, (2012). NIH Image to ImageJ: 25 years of image analysis. Nature Methods, **9**, 671–675.

**Figure S7**


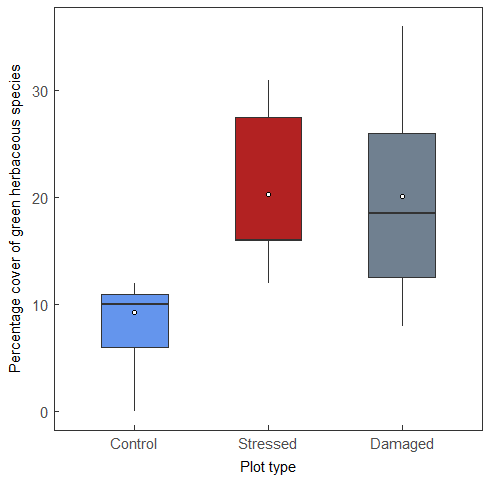


Fig. S7: Percentage cover of green herbaceous species across the growing season in control, stressed and damage plots. There was significantly higher percentage cover of herbaceous plants in both stressed and damaged plots compared to controls (F=11.1, d.f.=2, 47, p<0.001, Tukey HSD: p<0.05).
